# Classification of behaviour with low-frequency accelerometers in female wild boar

**DOI:** 10.1371/journal.pone.0318928

**Published:** 2025-02-26

**Authors:** Thomas Ruf, Jennifer Krämer, Claudia Bieber, Sebastian G. Vetter

**Affiliations:** Department of Interdisciplinary Life Sciences, Research Institute of Wildlife Ecology, University of Veterinary Medicine, Vienna, Austria; University of Ferrara Department of Life Sciences and Biotechnology: Universita degli Studi di Ferrara Dipartimento di Scienze della Vita e Biotecnologie, ITALY

## Abstract

Accelerometers with low sampling rates (1 Hz) are commercially available as ear tags. While an automated and therefore undisturbed sampling of animal behaviour can be useful not only in behavioural studies but also in ecological or wildlife management studies, the usefulness of such ‘a low data collection rate for the prediction of behaviours was the key question addressed here. We classified the behaviour of female wild boar, kept under semi-natural conditions in a large outdoor enclosure, using acceleration data. Predictions were based on a machine learning algorithm, specifically a random forest model in the open software h2o. Remarkably, prediction of many behaviours was possible using ear-tag acceleration sensors that sampled data only at a low frequency. This measurement device was mainly used to minimise the potentially harmful effects caused by the repeated capture of wild animals to exchange batteries. Long battery life will also help to collect long-term accelerometer data and has the potential to explore seasonal and inter-annual trends. Foraging, lateral resting, sternal resting and lactating were identified well, scrubbing, standing and walking not reliably. Balanced accuracy depended on the behaviour type and ranged from 50% (walking) to 97% (lateral resting). Results show that static features of unfiltered acceleration data, as well as of gravitation and orientation filtered data, were used in the prediction of behaviour. The waveform of certain behaviours in the sampled frequency range played no important role. Certain positively identified behaviours, such as food intake and lactation, could be of interest for wildlife managers attempting to control population growth in this pest-species. We provide several R-scripts that allow the analysis of behavioural accelerometer data.

## Introduction

Accelerometers have been deployed in many species over the last decades to quantify animal behaviour. The purpose of acceleration measurements, in up to three axes, has been to identify animals as active or resting, to compute proxies of their energy expenditure, or to classify animal behaviour as foraging, running, etc. [e.g., 1–4]. Identifying behaviour from body acceleration requires advanced analysis techniques such as principal principal component analysis, discriminant analysis or, often used more lately, machine learning (ML) algorithms. The advent of ML has very much facilitated the use of accelerometer data to classify behaviour [5, 6].

Acceleration was frequently used among non-human mammals, mainly in domesticated and captive animals with a roughly even split between domesticated/captive and wild animals [1]. There is great interest in classifying behaviour of wild, possibly free-living species [e.g., 7–9]. Because the waveform of acceleration data is often characteristic for a behavioural pattern, acceleration should be collected with a sampling rate high enough to allow the resolution of even high frequencies. This is due to the so-called Nyquist frequency. The highest frequency that can be detected in any signal without distortion is one half of the sampling rate [10]. In other words, it is the frequency whose cycle-length is twice the interval between samples. Thus, if acceleration is sampled for example at 10 Hz, Fourier analysis, which is routinely used to quantify its waveform, is limited to frequencies slower than 5 cycles per second. Although in the past sampling rates of accelerometers were sometimes as low as 0.5 Hz, in the majority of studies reported it ranged from 8 to 100 Hz [review in 1]. In a recent review on the use of accelerometers in humans Farrahi indeed recommended to use a sampling rate of 20–30 Hz to predict the type of activity. There are few studies that used low sampling rates [11]. In fact, accelerometer measurements are typically collected at very high resolution (>10 Hz).

The problem with high sampling rates is that they are obviously energy consuming, costly, and limit the long-term use of acceleration sensors without recharging. Whereas human subjects are typically cooperative in exchanging the battery of devices, wild animals are not. Repeatedly recapturing wild species to exchange logger batteries can lead to severe stress [12]. In the worst case a condition called capture myopathy, a consequence of extreme stress, can even lead to the death of animals [13]. On the other hand, important research questions, such as seasonal investigations or even studies on the life history of animals often require long-term studies.

For example, we faced this problem studying energetics in female wild boar, *Sus scrofa*. As this mammal is highly seasonal, with energy turnover being highest in winter, we recorded its heart rate, body temperature and behaviour for two consecutive years or more [review in 4]. We restricted recapture of the wild animals, kept in a large outdoor enclosure, for the purpose of logger and earmark exchange to once per year. This was only possible using a 1 Hz sampling rate for 3D acceleration data as higher frequencies would have depleted the ear tag battery too quick. Another advantage of a low sampling interval were lower transmission costs of the data to our server, which had to be facilitated via the mobile network. Here, we attempted to see whether certain behaviours, and if so which, of female wild boar can be identified using a sampling rate of 1 Hz only. Theoretically, the type of acceleration recorded with these devices should only allow the prediction of behaviours associated with relatively slow frequency characteristics.

In short, it turns out that we were able to predict various behaviours, especially foraging and resting in different body positions with an overall accuracy of 94.8%. However, the model also predicted less frequent behaviours, such as lactation, with high reliability. This was achieved by a ML model, employing a random forest (RF) algorithm. This model was built using the “h2o” open-source project called from within the free software R.

Here, we used and provide several R scripts (which can be employed for any accelerometer frequency) to either build a new model from paired acceleration-behaviour data, or to predict behaviour from an existing model, as provided in this case for wild boar. These scripts make it particularly easy to change time-windows for the underlying learning algorithm.

## Methods

Ethics The present study was discussed and approved by the ethics and animals’ welfare committee of the University of Veterinary Medicine, Vienna, Austria, in accordance with good scientific practice and national legislation (GZ: BMWFW-68.205/0151-WF/V/3b/2016 and GZ: BMWFW-68.205/0224-WF/ V/3b/2016). All methods were carried out in accordance with relevant guidelines and regulations. We confirm that the study was carried out in compliance with the ARRIVE guidelines [14].

### Animals and study area

The study animals were kept in an outdoor enclosure (~55 ha) in Austria. The enclosure was part of a game reserve, which was enclosed by 2.5 m high, solid, non-transparent fencing and was closed for the public. Thus, the study site provided an environment without disturbances due to hikers, bikers or straying dogs. There were no battue hunts or other disturbances due to hunting or forest management activities during the study period in the enclosure. The study enclosure was covered with a deciduous forest, mainly Turkey oak (*Quercus cerris*) and pubescent oak (*Quercus pubescens*) and included only few meadow patches. For the present study 13 adult females were used. We concentrated on females only because the live capture and handling of males are hampered by the large size and ferocity of boars and would have been much more time-consuming. Note that animals were not only captured but handled without anaesthesia, for example during weighing. Moreover, we are not aware for any sex-specific behaviours, if not sexual behaviour itself, of wild boar [e.g., 15].

During the study period (28/02/2017–22/05/2017), animals were supplemented with 1–1.5 kg corn per individual once a day at two feeding pens, each ~40×20 m, which could be entered and exited through two different entrances by the animals, respectively, and which were also used to capture the animals in autumn (e.g., for weighing or change of ear tags). This supplement may affect the interpretation of foraging but not of other behavioural states. The feeding pens were equipped with a telemetry system (Smartbow GmbH Austria, 2018) that also covered the surrounding of the pens consisting of a total of 10 receivers and telemetry ear tags (34 g; 52 mm x 36 mm x 17 mm) collecting 3D- acceleration and temperature data at 1 Hz. The transmission from ear-tags to receivers was achieved through a wireless local area network (WIFI). 3D-acceleration data were transmitted via a solar powered network interface to a server for storage.

Before applying telemetry ear tags, each wild boar was identified by a numbered and coloured ear tag (left ear), a RFID (radio-frequency identification) ear-tag (right ear), or, if both have been lost, by scanning the RFID implant (all three applied in a previous project; see [16]). After removing the old RFID-ear tag in the right ear, the telemetry ear tag with a unique ID number and MAC-address (medium access control address; i.e., a unique hardware identifier) was applied in the same place with special pliers. From the triaxial accelerometers in the ear tags we calculated overall dynamic body acceleration (ODBA) according to Wilson [17] using a time window for smoothing of 3 s. The average ODBA, i.e. the overall acceleration, was quite typical for each behaviour, e.g., ~50 for resting in sternal position (RSP) but >500 for scrubbing (Table 1).

**Table 1:** Description of the behaviours that were used to label the video records of wild boar. Recoding of 5.34% of the data (4:42:19 hours of individual wild boar behaviour) showed a high interobserver reliability (unweighted Cohens Kappa = 0.83). The rightmost column shows mean ODBA ± standard error of the mean (SEM).

Behaviour	Description	ODBA ± SEM
RLP (Resting in lateral position)	Wild boar is resting on its side. All four legs are spread out to one side. No breastfeeding is taking place.	77.13 ± 3.37
RSP (Resting in sternal position)	Wild boar is resting on its chest and abdomen. Legs are either underneath the body or spread out to the front and/or back.	52.52 ± 2.02
Foraging	Wild boar is standing or walking slowly while the snout is touching the ground or submerged into the ground. The snout is used to dig into the ground. Terminated if the snout does not touch the ground for up to 3 seconds.	391.88 ± 0.77
Lactating	Like resting in lateral position but the wild boar is breastfeeding its juveniles. Lasts as long as one or more juveniles are sucking at the teats of the wild boar.	137.35 ± 5.38
Scrubbing	Scrubbing of head, torso or ear while the wild boar is standing. A tree stump or something similar is used to scrub against.	553.26 ± 28.60
Standing	All four legs stand on the ground, the pair of front legs and the pair of hind legs is parallel. The weight is spread evenly on all four legs.	168.64 ±3.59
Walking	Four cycles, legs are being moved in parallel, three feet always touch the ground. Rather slow. At least 3 steps have to be made to be counted.	447.67 ± 3.69

Animals were accustomed to the presence of observers for approximately one month prior to video recordings to ensure they could be observed not only while in the pen, but also in the surrounding area. This was done by two observers being present in and around the feeding pen on six days a week for about five hours per day on average around the time of feeding (early afternoon). As the wild boars were used to the presence of human observers already from previous years this was sufficient to re-accustom the animals and assure high quality video recording of a large range of behaviours including behaviours that animals would not show when stressed like resting in lateral position or even lactation. For videos recording a handheld camera was used. Behaviour of several wild boars could be recorded at the same time. Video recordings of the behaviours were taken from outside of the feeding pen and the direction of filming was always chosen to ensure that the highest number of wild boar tags could be filmed at once. During the recording animals were named and the position of each wild boar in the frame was regularly described verbally. Whenever a wild boar entered or left the frame it was announced with direction the animal was coming from or going to and the colour-number identification code. Naming was done as often as possible but at least every time a wild boar entered, left or re-entered the frame. The exact time and date of each video was needed to be able to pair them with the acceleration data. To make sure time was correct a radio-controlled clock with a digital display of date, hour, minutes, and seconds was held into the camera frame every half hour for at least five seconds. With this time the behaviours could be merged with acceleration data, for which timestamps were retrieved from network time which was synced once a day from the internet.

### Behaviour types recorded in *Sus scrofa*

Foraging, walking, standing, resting in sternal position, resting in lateral position, scrubbing, and lactating behaviours were coded from video tapes at 1 Hz according to their definition (Table 1) to match the sampling frequency of accelerometers (i.e., in the video coding program the exact start time of each behaviour was noted, automatically ending the previous behaviour, and the data subsequently exported from the video coding program listing the behaviours in 1 Hz timestamps; behaviours lasting less than a second were ignored). To analyse these video-records we used the labelling software Solomon (Solomon Coder beta 17.03.22). Videos were usually watched in real time during analysis, except when the wild boar was showing a static behaviour. Each video was watched individually for each animal. If there was any uncertainty of which wild boar could be seen data were not coded.

Data frames derived from the program Solomon were imported into the program R and merged with respective 3D-acceleration data. Additionally, acceleration data were transformed using the so-called jerk filter [18]. This transformation provided data that showed the change of acceleration instead of the total acceleration. These resulting data were independent from orientation and from gravitational pull that could not be used as earmarks were freely movable. Change of the angle of acceleration was used to calculate directional changes.

All models to predict behaviour from acceleration data were built using the R interface to h2o.ai, both are free, open-source software packages. The models were always computed using a randomly assigned 50% of the data for training, 25% for validation, and 25% for testing. We verified that all behaviour classes were present in all of the three data-subsets. All metrics of model performance, such as sensitivities, were computed using the R-package ‘caret’ [19]. Please note that in caret the predicted and actual classes are reversed in the confusion matrix.

**Fig 1:**
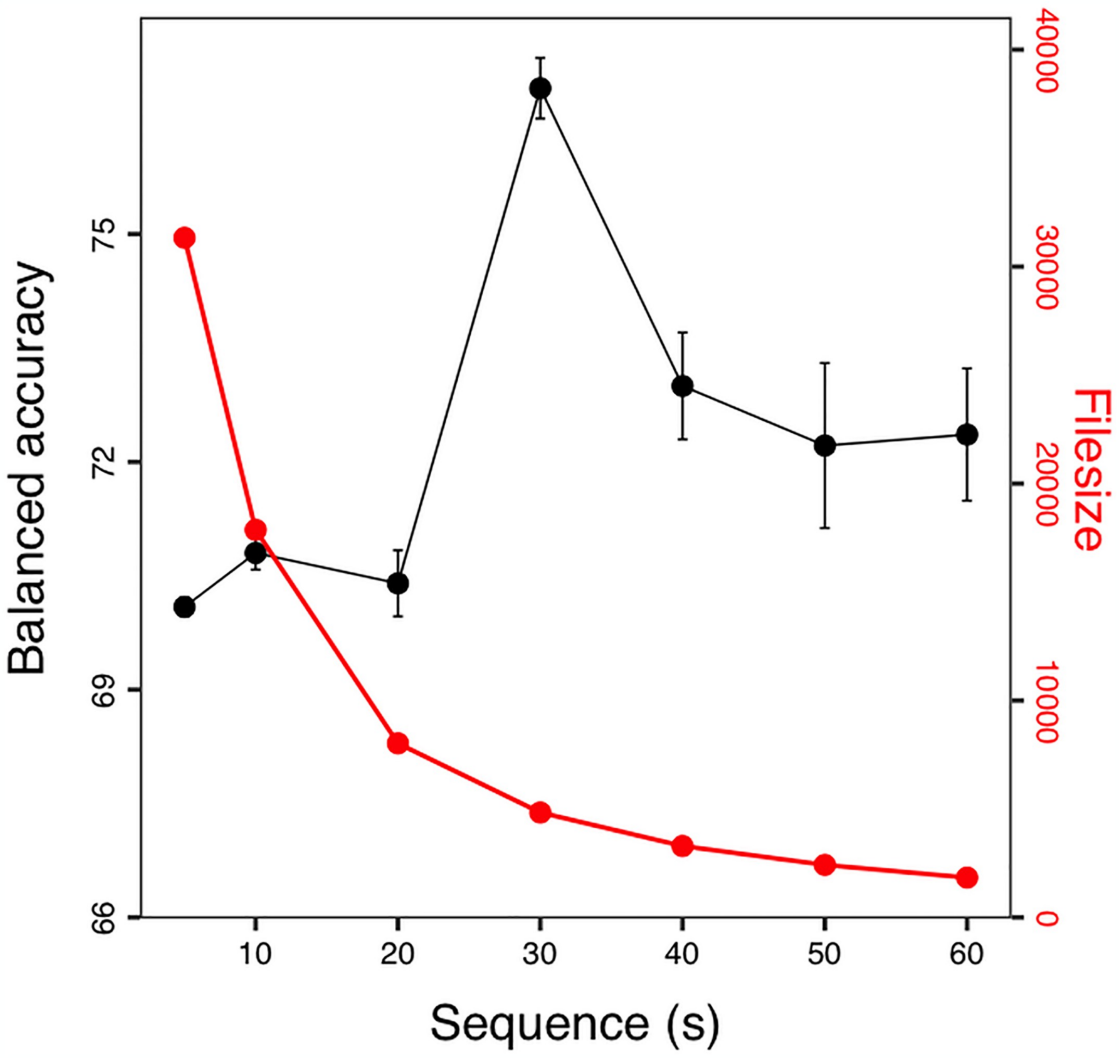
Time window size. The effect of window size on balanced accuracy of the RF model (black; mean ± SD) and the underlying file size (red; lines of data). Overlapping data windows were increased from 6 to 60 s.

The variables used for ML were calculated for time windows of different lengths (i.e., 6, 10, 20, 30, 40, 50, 60; see Fig 1). Inevitably occurring terminal time periods at the end of a behavioural sequence of a given animal that were shorter than the time window length were discarded. The moving time windows had a 50% overlap to prevent an overestimation of the classifier performance [20]. For each time window, we calculated the 11 major moments (such as mean, kurtosis etc.; R package moments [21] as well as the power of the main frequencies (i.e., estimated spectral densities obtained from Fourier analysis of the time series) in the acceleration variables (both filtered and unfiltered) used for ML. The behaviour that occurred the most within a time window was set as the behaviour of the respective time window. This procedure was followed to resemble the applied situation when behaviours are not known a priori.

The variables computed from acceleration data were fed into ML algorithms, either an artificial neural network (ANN) or a random forest (RF) algorithm. The only value changed from the default was the maximum number of trees which was increased from 50 to 100 in RF models. The relative importance of variables was computed in h2o according to Gedeon [22].

## Results

The length of recorded behaviour per wild boar data ranged from 0.04 h to 9.5 h (mean: 3.1 h). The total length of behavioural data for which acceleration data were evaluated was 40.8 h. Not unexpectedly, the behaviours in the vicinity of the feeding station were 80.9% foraging, 5.9% walking, 4.1% RSP, 4.1% standing, 1.5% resting in lateral position (RLP), 1.2% lactating and 1.3% other. Behaviours were not evenly distributed with lactating, RSP, and RLP occurring only in 7 out of 13 animals.

Each behaviour was associated with a typical mean ODBA (Table 1), i.e., ODBA values tended to be within a consistent and distinct value range for each behaviour. Thus, it provided the opportunity to measure the intensity of activity. However, adding ODBA to the predictors in the model did not improve behaviour classification, which is why it was left out.

Using ANN was quite successful, with 2 layers of 225 neurons each we reached an overall accuracy of 92.3% (see ANN.R in S1 File). The RF algorithm was even more appropriate reaching an oval accuracy of 94.8% (95% CI: 93.3–95.9%). This was mainly because the proportion foraging was the most dominant behaviour and was identified well. Therefore, it seemed appropriate to use the mean balanced accuracy, which is unbiased by class frequency, as a measure of model performance. The balanced accuracy increased with sequence duration and reached an optimum of ~77% at a 30 s window, when compared for identical test data (Fig 1). Model performance did not change much as the time window was further increased to 60 s. Simultaneously, the data base with uninterrupted behavioural observations (i.e., time windows) declined rapidly (Fig 1). Therefore, a model based on 30 s data (with 15 s increments) seemed best.

Confusion matrix of an ML model to predict behaviour from acceleration data, as validated by video recordings of wild boar. The correct predictions are in bold face. The degree of wrong predictions is shown in the columns Error and Rate. The overall accuracy of the model was 1–0.052 = 1-total error = 0.948. Rare behaviours (in the dataset), like scrubbing were never predicted by the model.

Sensitivity is a measure of how well the model can identify true positives and specificity is a measure of how well it can identify true negatives. Their mean, balanced accuracy, is a measure of performance in the imbalanced class setting.

Certain behaviours could be identified well by acceleration data, others not reliably at all. A confusion matrix for the best machine-model is given in Table 2. The best predicted behaviour was foraging, followed by RLP, RSP and lactation. Some behaviours, like scrubbing, were extremely rare and could not be predicted reliably. For example, “walking” occurred 25 times but was mistaken for “foraging” every time (Table 2). Those rare behaviours may be entirely omitted. The sensitivity, specificity and balanced accuracy for the behaviours is given in Table 3.

**Table 2:** Confusion matrix of the best RF model.

**Actual Behaviour**	**Predicted Behaviour**									
		RLP	RSP	Foraging	Lactating	Scrubbing	Standing	Walking	Error	Rate
	RLP	**33**	0	1	0	0	1	0	0.057	2/35
	RSP	1	**62**	5	1	0	3	0	0.139	10/72
	Foraging	0	0	**1013**	0	0	4	0	0.004	4/1017
	Lactating	1	2	1	**13**	0	0	0	0.235	4/17
	Scrubbing	0	0	2	0	**0**	0	0	1.000	2/2
	Standing	1	2	12	0	0	**17**	0	0.468	15/32
	Walking	0	0	25	0	0	0	**0**	1.000	25/25
	Totals	36	66	1059	14	0	25	0	0.052	62/1200

**Table 3:** Sensitivity and specificity (% accuracy) computed from the best model.

	RLP	RSP	Foraging	Lactating	Scrubbing	Standing	Walking
Sensitivity	94.28	86.11	99.61	76.47	0.00	53.12	0.00
Specificity	99.74	99.64	74.86	99.91	100	99.31	100
Balanced Accuracy	97.01	92.87	87.24	88.19	50	76.20	50

**Fig 2:**
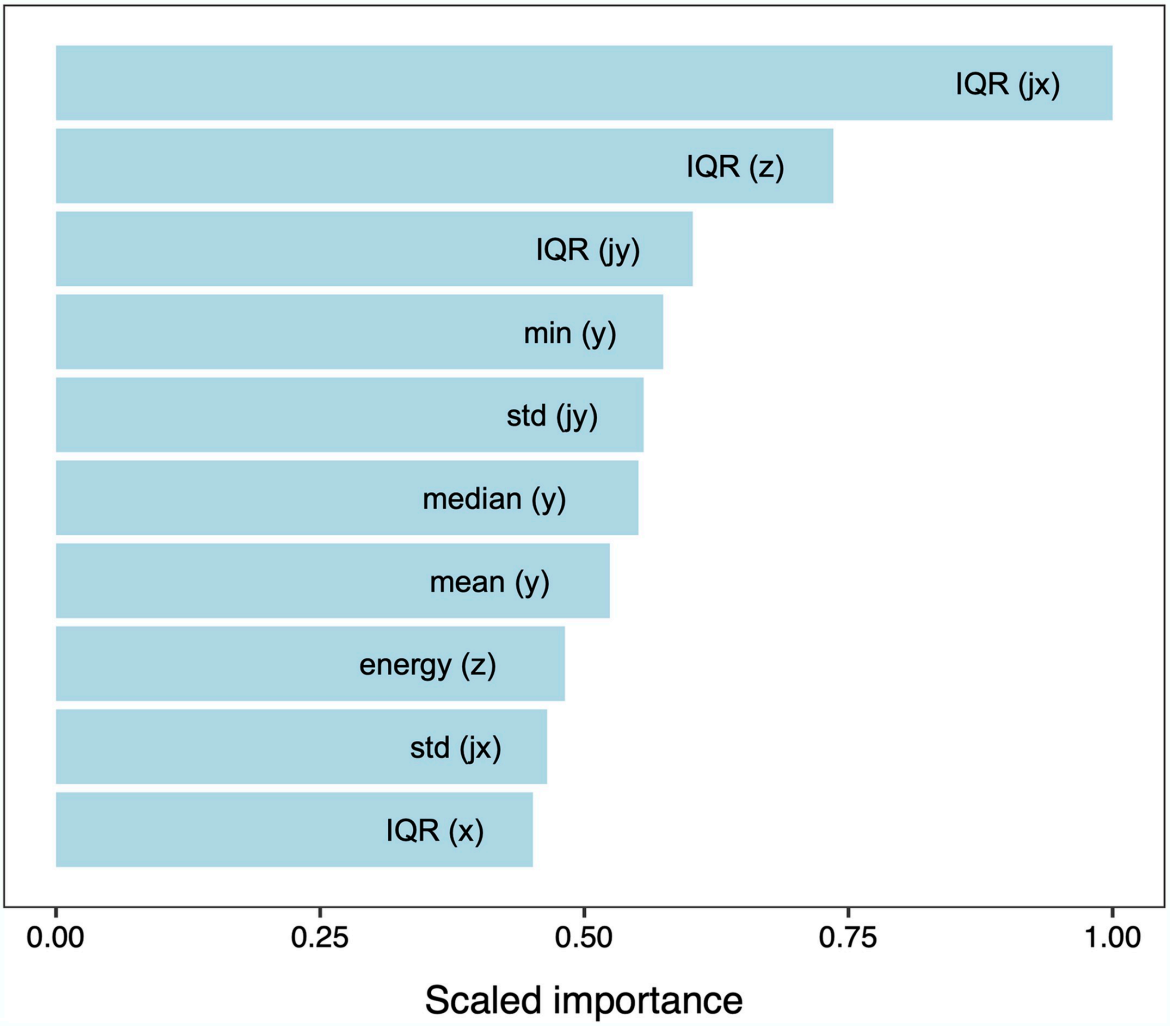
Variable importance. Relative importance for the 10 most important variables (top to bottom) in the best model predicting behaviour from acceleration. The three most important variables were all interquartile ranges. The labels in parenthesis indicate which of the accelerometer axes (x,y,z) was important, and whether it was jerk-corrected (j).

Both raw acceleration data and the additional jerking-filtered acceleration data were important in predicting a behaviour. This conclusion was based on the finding that 6 of the 10 most important variables were computed from acceleration data as such, while 4 were computed from jerking-filtered measurements. The three most important variables (scaled importance 0.60–1.00) were all interquartile ranges (Fig 2), and the first variable that was the power of a frequency was ranked 52, i.e., completely unimportant (scaled importance 0.07).

## Discussion

Here, we successfully employed a RF algorithm to predict behaviour, a model that outclassed artificial neural networks. Both algorithms have been used in the past to classify behaviour [23]. The rate of correct predictions in the present study was improved by the size of the video basis. The positive identification of a certain behaviour (its sensitivity) was correlated with its commonness (r = 0.76; Spearman’s coefficient).

The type of behaviour observed was clearly biased towards foraging, which was expected in a feeding enclosure. Both the directly observed amount of foraging (80.9%) and the predicted foraging (88.2%) were much higher than expected from other models, also using wild boar [24]. This is not surprising, because behavioural observations were made in a large feeding pen. However, the main purpose of this study was not to establish a representative behaviour pattern for wild boar but methodological namely to see if a ML model would predict behaviour with sufficient accuracy if acceleration measurements are slow (1 Hz). This was clearly the case. Our model identified behaviour with an accuracy of ~95% and mean balanced accuracy of all behaviours was ~77%.

Whereas boar in an enclosure may show shifts in the frequency of behaviours shown, there is nothing to suggest the occurrence of unnatural behaviours in captive wild boar. Unfortunately, there are no behavioural records from free-living animals for comparison.

We have reason to believe (see below) that certain behaviours, like scrubbing, were simply too rare to be learned reliably. These behaviours could be removed from the dataset, to facilitate the focus on certain important variables, such as lactation. Alternatively, rare behaviours might be identifiable using more frequently sampled acceleration values or maybe observed over a longer time-period or in a different setting, i.e., outside a feeding pen. This might for instance be the case for walking, which by our algorithm could not be differentiated properly from feeding. This was most likely because the two behaviours were too intermixed with each other and many time windows identified as feeding contained partly also the behaviours walking and/or standing but were identified as feeding as this was the most frequent behaviour in the given time window. This is reflected by the fact that walking made up 5.9% of the total observation time but was categorised in only 2% of time windows (25/1197). Following a slightly different procedure and training, the algorithm with time windows only containing one behaviour, respectively, potentially could have resulted in a better prediction also for behaviours that often occurred intermixed with other behaviours like walking and standing. On the other hand, this would have reduced the number of time windows available for training essentially and potentially resulted in an overall decreased performance.

Of course, a weakness of the supervised method used is that certain behaviours such as wallowing or swimming were not represented in the training data. These behaviours may be particularly important for thermoregulation in wild boar, which could be an additional driver of population growth under global climate change. This underlines the need for better data signals that contain nearly all representative behaviours [24]. Another possible limitation might seem to be the location of the accelerometer which was mounted freely rotating to the ear of the animals. Indeed, other more rigid positions like fixating it to the leg of the animals or on a collar potentially could have resulted in accelerometer data even better suited to predict wild boar behaviour. These positions however imply a high danger for the sensors to be damaged either by the animal itself or by other sows [18]. The shape and large diameter of the neck does further not allow a save fixation of a collar, especially for the long-term data recording. Therefore, applying the sensor to the ear of the animal is the most desirable solution for the practicable application of accelerometers to sows [25]. Also from ethical aspects (e.g., low risk of infection) the ears seem the best position for an acceleration logger.

The sensitivities and specificities of resting in the lateral and sternal position were very high (Table 3) which is in accordance to findings in domestic sows [2]. Surprisingly, we found that lactating of females could be predicted in ~76% of all cases (Table 2), although it should be very similar to resting in a lateral position. However, ODBA during lactation was about twice as high as during resting (Table 1), indicating that suckling young generate substantial gravitational forces.

Giving milk to young is a social interaction that indicates successful reproduction without direct observation. This finding may be of interest to wildlife managers that are trying to control population growth in this species. Wild boar is a pest species with populations increasing worldwide and substantial damage to crops or endemic plants [23, 26]. Thus, an early sign of reproduction may help to control population expansion in this species. Lactation, I.e., RLP with a high simultaneous movement not only indicates the presence of young, but high investment in their growth [e.g., 27]. At a minimum lactation behaviour will provide managers with an estimate of the proportion of females with successful reproduction. When entered, for instance, into Leslie matrix models, they will inform wildlife managers if a population can be expected to grow or decline. Accordingly adjusted management measures may result [28]. The same potentially holds true for foraging, provided it is correlated with actual food uptake. Food availability is the most important driver of population increase in wild boar [23, 26] and information on the time spend feeding may be highly valuable for wildlife management.

Further insights on the behaviour criteria used for classification can make use of the computed variance importance. The three most important variables were all interquartile ranges (IQRs), that is, a measure of both the central tendency and variation in acceleration. This variable was most characteristic and was crucial for the RF algorithm. For example, the mean IQR was only 36.2 in RSP but 553.3 in scrubbing data. The fact that the mean IQR was clearly higher resulting from walking and scrubbing than any other behaviour (≤460) reinforces our assessment that these behaviours were only difficult to classify because they were rare. Note that including ODBA did not improve the model.

Also, the fact that frequencies were unimportant variables explains how the low-speed accelerometers used were able to lead to a high overall accuracy of prediction: The most important information was contained in static characteristics, such as the IQR. Its waveform, especially the power of frequencies above 0.5 Hz played no role in predicting behaviour. This is not to say that lower periods may well be characteristic for behaviours obtained with accelerometers that are able to sample at higher rates.

We already provided information on the cost of an ear tag system. However, a purely economic comparison between using slow and fast accelerometers would not be sensible. First, Smartbow ear tags, as used here, are commercially available only in the slow 1 Hz version, higher sampling rates are restricted to scientific studies only [29]. Importantly, the monetary cost of accelerometers is negligible compared with the necessary data transmission system or personnel costs of a project. Much more significant are the costs of a high sampling rate system in terms of battery life and animal welfare. Battery life almost linearly scales with sampling frequency and is, for instance, more than halved when the logging sampling rate is decreased from 100 Hz down to 25 Hz [30]. Lowering the sampling rate thus increases the total observation period decreases the need for frequent recapturing and immobilizing wild animals, which may be even fatal. Of course, there is generally a trade-off between sampling rate and accuracy of behavior detection [e.g., 2, 11, 31]. Only certain species display short bursts of special movements that indeed require high sampling rates [e.g., 31–33]. Therefore, the choice of collection speed should include considering a species behavioural characteristics.

Nowadays, software is available for the Input files of ML algorithms such as the R-package rabc [31]. However, we used and provide four R-scripts designed to ease the process. Script A (“make jerked”; S1 File) is a short R-script that adds the jerk-filtered data to a table with animal name(s) plus acceleration data. If the purpose is the generation of a new behavioural model, the user has to add a column “behaviour” with the corresponding behaviour observed. If the goal is prediction of behaviour from acceleration and an existing model the user should also generate a column “behaviour” but assign it the label “unknown”. Script B (“make input file”; S1 File) will then be used to generate an input file with variables for ML, given a certain time window size. The same script can be used to generate input files for a new model (script C) or to predict behaviour (script D). Given the 50% overlap of moving time windows we chose here, the time interval used for generating the variables for ML is computed in script B from half the window size. Script B may be used as is for other sampling frequencies and may be slightly modified to make the time interval and window size mutually independent (i.e., use overlaps of moving time windows different from 50% overlap). It also includes a routine to compute the Fourier spectrum from time series of acceleration data. Script C (“build model”; S1 File) is used to build a model based on h2o.randomForest. Use h2o.deeplearning for an ANN algorithm, depending on the data (Script E”ANN”, S1 File). Script D (“prediction”; supplementary material) is used to predict behaviour from a model loaded from disk. The supplementary material also includes a binary file including several example data files, the data file for the 30 s time window, as well as the original behavioral data (S1 Data).

## Conclusions

Despite the low frequency acceleration sampling (1 Hz) even infrequent behaviour of wild boar could be predicted with high sensitivity and specificity, for many behaviours. This was possible because the underlying RF algorithm used mainly static variables, such as the IRQ of acceleration data to classify behaviour. These data may be used by wildlife managers to develop appropriate measures against this or other pest species.

## Supporting information

S1 File All R-Scripts used in the analysis including the scripts to A) create jerk.-filtered data (make-jerked.R), B) create the input file for the analysis (make input file.R), C) build the random forest model (build model RF.R), D) predict behaviors (prediction.R), and E) to run the artificial neural network (ANN.R).
(DOCX)

S1 Data Binary file to be loaded in R containing two example data files, the data file for the 30 s time window, as well as the original behavioral data. (RDATA)

## Abbreviations

ANN: Artificial Neural Network
ML: Machine Learning
RF: Random Forest
RLP: Resting in Lateral Position
RSP: Resting in Sternal Position
