# Supplementary material for: Classification of behaviour with low-frequency accelerometers in female wild boar
Source: PLoS One. 2025 Feb 26;20(2):e0318928. doi: 10.1371/journal.pone.0318928 (PMC11864551; doi:10.1371/journal.pone.0318928)
Supplement: S1 File — All R-Scripts used in the analysis including the scripts to A) create jerk.-filtered data (make-jerked.R), B) create the input file for the analysis (make input file.R), C) build the random forest model (build model RF.R), D) predict behaviors (prediction.R), and E) to run the artificial neural network (ANN.R). (DOCX) [file pone.0318928.s001.docx]

**A) make-jerked.R**

dat=read.csv("Anne.csv") #Example file

source("JerkingVectorized.R") #load routine

data=data.frame(dat$accX,dat$accY,dat$accZ)

jerk=JerkingVectorized(data) #apply it

joined=cbind(dat,jerk)

head(joined)

joined$behaviour="unknown"

write.csv(joined,file="AnneJ.csv",row.names = F) #store new data

**JerkingVectorized.R**

JerkingVectorized <- function(Data){

x.axis <- Data[,1]

y.axis <- Data[,2]

z.axis <- Data[,3]

numerator <- (x.axis[-length(x.axis)]*x.axis[-1]) +

(y.axis[-length(y.axis)]*y.axis[-1]) +

(z.axis[-length(z.axis)]*z.axis[-1])

denominator <- (sqrt((exp(x.axis[-length(x.axis)])) + (exp(y.axis[-length(x.axis)])) + (exp(z.axis[-length(x.axis)])))) *

(sqrt((exp(x.axis[-1])) + (exp(y.axis[-1])) + (exp(z.axis[-1]))))

angle <- numerator/denominator

angle <- acos(pmin(pmax(angle,-1.0),1.0)) * (180/pi) #convert cosine value to radians and then to angles

jerk_x <- x.axis[-1] - x.axis[-length(x.axis)]

jerk_y <- y.axis[-1] - y.axis[-length(y.axis)]

jerk_z <- z.axis[-1] - z.axis[-length(z.axis)]

jerk_x <- c(((1+(angle/180))* jerk_x),NA)

jerk_y <- c(((1+(angle/180))* jerk_y),NA)

jerk_z <- c(((1+(angle/180))* jerk_z),NA)

return(data.frame(jerk_x, jerk_y, jerk_z))

}

**B) make input file.R**

win=30 #Change window size

si=1 #Example sampling interval

#inp="AnneJ.csv" #Prediction example animal Anne

inp="BehavVal.csv" #Data used to make model

#########################################################

dat=read.csv(inp)

dat=as.data.frame(dat)

library(moments)

rspec=function (x,sampint=1,from=NULL,to=NULL,spans=NULL,plotit=F){ #compute Fourier spectrum

x.spec <- spectrum(x,log="no",spans=spans,plot=F)

spx <- x.spec$freq/sampint

spy <- 2*x.spec$spec

if (is.null(from)) from=min(spx)

if (is.null (to)) to=max(spx)

ix=which(spx>=from & spx<=to)

spx=spx[ix]

spy=spy[ix]

if (plotit==T) plot(spy~spx,xlab="frequency",ylab="spectral density",type="l",xlim=c(from,to))

return(spy)

}

ff=function(x){ # compute all moments for each time series

x=na.omit(x)

m=mean(x)

me=median(x)

sd=sd(x)

kt=kurtosis(x)

sk=skewness(x)

mi=min(x)

mx=max(x)

e=sum(x^2)/length(x)

ir=IQR(x)

ar=acf(x,plot=F)[[1]][2:3]

return(c(m,me,sd,kt,sk,mi,mx,e,ir,ar,rspec(x)))

}

ix=which(duplicated(dat$time)) #remove duplicates

dat=dat[-ix,]

dat$time=as.POSIXct(dat$time)

dat$dtime=difftime(dat$time,min(dat$time),units="secs")

dat$dtime=as.numeric(dat$dtime)

dat$time=as.numeric(dat$time)

block=1

count=0

dt=diff(dat$dtime)

dt=c(0,dt)

for (i in 1:length(dat$time)){

if (dt[i]>si) count=count+1

block[i]=count

}

block=paste(dat$name,block)

pb = txtProgressBar(min = 0, max = 100, initial = 0)

stp=(round(win/2))

stp2=floor(stp/2)

m=b=tme=nme=NULL

new=split.data.frame(dat,block) #split according to animal, and uninterrupted sequence

for (i in 1 :length(new)){

perc=(i/length(new)*100)

setTxtProgressBar(pb,perc)

data=new[i]

data=as.data.frame(data)

names(data)=names(dat)

if (length(data$name>0)){

intervals=seq(1,length(data$name),by=stp)

for (j in 1:length(intervals-1)){

sect=data[intervals[j]:(intervals[j]+win-1),]

sect=na.omit(sect)

if (length(sect$name)==win) { # Change here to also get shorter chunks

res=c(ff(sect$accX),ff(sect$accY),ff(sect$accZ),

ff(sect$jerk_x),ff(sect$jerk_y),ff(sect$jerk_z)) #compute moments for each series

tt=sect$time[1+stp2]

bt=table(sect$behaviour)

nm=sect$name[1]

v=names(bt)[which.max(bt)]

tme=c(tme,tt)

nme=c(nme,nm)

m=rbind(m,res)

b=c(b,v)

}

}

}

}

close(pb)

datneu=data.frame(nme,tme,b,m)

repl=paste("-",win,".inp",sep="")

name=sub(".csv",repl,inp)

write.csv(datneu,name,row.names=F) #make new name and store data

print(paste("Stored",name))

**C) build model RF.R**

library(h2o)

lh2o=h2o.init(nthreads=-1, max_mem_size="2G")

h2o.removeAll()

df <- h2o.importFile(normalizePath('BehavVal-30.inp'))# get input frame

ix=which(names(df)=="tme") # remove if present

if (length(ix)>0) df=df[,-ix]

ix=which(names(df)=="nme") # remove if present

if (length(ix)>0) df=df[,-ix]

splits <- h2o.splitFrame(df, c(0.5,0.25), seed=1)

train <- h2o.assign(splits[[1]], "train.hex") # 50%

valid <- h2o.assign(splits[[2]], "valid.hex") # 25%

test <- h2o.assign(splits[[3]], "test.hex") # 25%

response <- "b"

predictors <- setdiff(names(df), response) #make response and predictors

#make rf model

mrf<- h2o.randomForest(x = predictors, y = response,

training_frame = train,

validation_frame=valid,

nfolds = 5,

ntrees=100,

seed=7) #make it repeatable

h2o.confusionMatrix(mrf, test) #make confusion matrix on test data

h2o.saveModel(object=mrf,path = "/Users/Ruf/wb/") #requires full path

**D) prediction.R**

inp="AnneJ-30.inp" #Change this to input file

##########################################################

library(h2o)

lh2o=h2o.init(nthreads=-1, max_mem_size="2G")

h2o.removeAll()

path="/Users/ruf/wb/DRF_model"

model=h2o.loadModel(path)

df <- h2o.importFile(normalizePath(inp))# get input frame

ix=which(names(df)=="b")

if (length(ix)>0) df=df[-ix]# remove colummn b, if any

df$preds=predict(model,df)[,1] #make predictions

df=as.data.frame(df)

df$preds=as.character(df$preds)

stp=min(diff(sort(df$tme)))[1]

stp2=(floor(stp/2))

win=floor(2*stp)

n=nchar(inp)

torepl=substr(inp,n-6,n)

orig=sub(torepl,".csv",inp)

odat=read.csv(orig)# read original file

ix=which(duplicated(odat$time))

odat=odat[-ix,]

odat$time=as.POSIXct(odat$time,origin="1970-01-01",tz="GMT")

odat$time=as.numeric(odat$time)

si=min(diff(sort(odat$time)))

odat$b_pred=" "

for (i in 1:length(df$preds)){

ix=which(odat$time %in% df$tme[i])

for (j in ix:(ix+stp)){

odat$b_pred[j]=df$preds[i] # assign predictions to time range

}

}

ix=which(names(odat)=="behaviour")

if (length(ix)>0) odat=odat[-ix]# remove behaviour if any

odat$time=as.POSIXct(odat$time,origin="1970-01-01",tz="GMT")

name=sub("inp","pred",inp)

write.csv(odat,file=name,row.names=F)

print(paste("Stored",name))

**E) ANN.R**

library(h2o)

lh2o=h2o.init(nthreads=-1, max_mem_size="2G")

h2o.removeAll()

df <- h2o.importFile(normalizePath('behav-2022-30.inp'))# get input frame

ix=which(names(df)=="tme")

if (length(ix)>0) df=df[,-ix]

ix=which(names(df)=="nme")

if (length(ix)>0) df=df[,-ix]

splits <- h2o.splitFrame(df, c(0.5,0.25),seed=16)

train <- h2o.assign(splits[[1]], "train.hex") # 50%

valid <- h2o.assign(splits[[2]], "valid.hex") # 25%

test <- h2o.assign(splits[[3]], "test.hex") # 25%

response <- "b"

predictors <- setdiff(names(df), response)

predictors

mb <- h2o.deeplearning(

training_frame=train,

validation_frame=valid, ## validation dataset: used for scoring and early stopping x=predictors,

y=response,

activation="Tanh",

epochs=50, ## hopefully converges earlier...

stopping_rounds=5,

stopping_metric="misclassification",

hidden=rep (225,2), ## default: 2 hidden layers with 200 neurons each,

variable_importances=T, ## not enabled by default

reproducible=T,

seed=7

)

h2o.confusionMatrix(mb, test)
